# Facile Growth of Cu_2_ZnSnS_4_ Thin-Film by One-Step Pulsed Hybrid Electrophoretic and Electroplating Deposition

**DOI:** 10.1038/srep19102

**Published:** 2016-02-23

**Authors:** Hung-Wei Tsai, Chia-Wei Chen, Stuart R. Thomas, Cheng-Hung Hsu, Wen-Chi Tsai, Yu-Ze Chen, Yi-Chung Wang, Zhiming M. Wang, Hwen-Fen Hong, Yu-Lun Chueh

**Affiliations:** 1 Department of Materials Science and Engineering, National Tsing Hua University, Hsinchu 30013, Taiwan; 2 Institute of Fundamental and Frontier Sciences, University of Electronic Science and Technology of China, People’s Republic of China; 3 Institute of Nuclear Energy Research, Tao-yuan, Taiwan, R.O.C

## Abstract

The use of costly and rare metals such as indium and gallium in Cu(In,Ga)Se_2_ (CIGS) based solar cells has motivated research into the use of Cu_2_ZnSnS_4_ (CZTS) as a suitable replacement due to its non-toxicity, abundance of compositional elements and excellent optical properties (1.5 eV direct band gap and absorption coefficient of ~10^4^ cm^−1^). In this study, we demonstrate a one-step pulsed hybrid electrodeposition method (PHED), which combines electrophoretic and electroplating deposition to deposit uniform CZTS thin-films. Through careful analysis and optimization, we are able to demonstrate CZTS solar cells with the V_OC_, J_SC_, FF and η of 350 mV, 3.90 mA/cm^2^, 0.43 and 0.59%, respectively.

The amount of solar energy we can potentially harvest is many times greater than what we currently consume and is therefore considered one of the most promising solutions to the energy crisis currently facing us in the twenty-first century^1^. Single junction inorganic solar cells with a maximum theoretical energy conversion efficiencies (η) of ~30% offers one such method to harness this energy^2^. To date, numerous technologies have been developed offering excellent performance, including crystalline Si, III-V compounds of GaAs and InP, Cu(In,Ga)Se_2_ (CIGS), and CdTe, all of which exhibit efficiencies over 20%^3^. CIGS-based solar cells currently hold the world record efficiency value at 21.7%, offering an attractive material candidate for thin-film solar cells^3^. However, their use of scarce and costly metals such as indium and gallium creates a bottleneck when considering low-cost electricity generation.

Cu_2_ZnSnS_4_ (CZTS) has been proposed as a replacement for CIGS due to its non-toxicity, use of relatively abundant elements in its composition and its excellent photo-electronic properties, including a direct bandgap of 1.5 eV^4^ and high absorption coefficient of over 10^4^ cm^−1^ ref. 4. There are numerous methods used to synthesize CZTS, including physically vacuum deposition techniques such as sputtering, evaporation, pulsed laser deposition (PLD), and chemically based non-vacuum deposition techniques such as electrodeposition, hydrazine-based solution and sol gel-based methods^5^. Among these chemically based non-vacuum deposition techniques, electrodeposition technique offers the advantages of low-cost, large area plating, in an industry compatible process, which is competitive with other techniques used for CZTS synthesis. Three primary electroplating based deposition techniques have been developed, including a stacked elemental layer approach (SEL), metal alloy electrodeposition (MAE) and quaternary electrodeposition (QED), each using ion based precursor species in the electrolyte^6^. Additionally, electrophoretic deposition (EPD) is an alternative approach whereby CZTS nanoparticles act as the precursor species in the electrolyte, typically enabling faster film formation^7^. In each case, a post deposition annealing step in the presence of a chalcogen source is required. Each process possesses their own merits and drawbacks, and all of them have drawn significant attention as suitable methods for large area CZTS thin film deposition.

Here, we propose a one-step pulsed hybrid electrochemical deposition method (PHED) to deposit quaternary elements of copper, zinc, tin and sulfur onto Mo coated soda lime glass (SLG) substrates. Unlike electroplating or electrophoretic deposition techniques that make exclusive use of ionic precursors or nanoparticles respectively, the proposed one-step PHED synthesis combines both techniques. Our PHED process combines the electroplating of Zn and Sn ions with electrophoretic deposition of a Cu_2−x_S nanoparticle precursor in a single electrolyte continuous process. This process offers a number of advantages over the individual methods. Firstly, we are able to avoid the time consuming pre-alloying/soft annealing inter-layer diffusion and interlayer rinsing steps associated with SEL approaches. Likewise, in comparison to MAE and QED, the formation of the Cu_2−x_S nanoparticles reduces the complexity of the electrolyte chemistry, offering better bath stability and a potentially faster overall deposition process. Finally, this method avoids the time consuming and complex process of CZTS nanoparticle preparation required when using an exclusively EPD deposition process^7^,^8^. In this work, we use a pulsed hybrid electrodeposition (PHED)^9^,^10^ method as opposed to a more conventional potentiostatic methods, which enables the formation of more uniform CZTS thin-films. A final sulfurization step is performed on the films by placing then in a sulfur vapor atmosphere for 1 hr. The materials characteristics and device performance were investigated in detail.

## Results and Discussion

**Figure 1:**
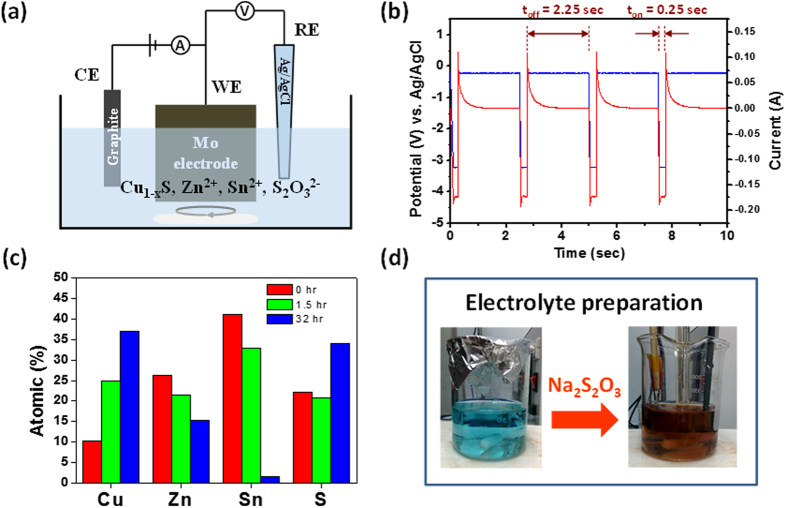
(**a**) A schematic of three-electrode cell used for pulsed hybrid electrodeposition. (**b**) Curve of voltage, current and timing utilised during pulsed electrodeposition. (**c**) Elemental compositions of PHED-deposited CZTS thin films prior to the sulfurization process with different complexation durations. (**d**) An optical image illustrating the change of color of the electrolytic solution after the addition of Na_2_S_2_O_3_.

Our PHED deposition of CZTS thin-films was carried out using a three-electrode cell, consisting of an Mo coated soda lime glass substrate as the working electrode (WE), a graphite bar as the counter electrode (CE) and a silver/silver chloride (Ag/AgCl) electrode as the reference electrode (RE). Figure 1(a) illustrates a schematic representation of the system with an electrolyte solution of deionized (DI) water containing 0.0022 M copper sulfate (CuSO_4_), 0.011 M zinc sulfate (ZnSO_4_), 0.005 M tin chloride (SnCl_2_), 0.08 M sodium thiosulfate (Na_2_S_2_O_3_) and 0.05 M tri-sodium citrate (Na_3_C_6_H_5_O_7_) used as a complexing agent for the Sn^2+^ ions^11^, respectively. The pH value of electrolyte was 5.81 with no further pH adjustment applied. To deposit the CZTS films, the pulsed electrodeposition method was applied using a 10% duty cycle (on-time duration of 0.25 sec at −3 V, off-time duration of 2.25 sec at 0 V) as shown in Fig. 1(b). The set potential of 0 V during the off-time is the potential difference between applied voltage and open circuit voltage, which is the steady state without any disturbance of applied potential. During the off-time, the relatively unstable as-deposited CZTS film will re-dissolve into electrolyte again, which will happen on the top part of film. This is beneficial to the film thickness uniformity. So we can observe that the current is still flowing to the positive direction during the off-time. The off-time allows the distribution of the precursor species within the electrolyte to return to a homogeneous state, thus allowing a higher deposition rate during the on-time when compared to potentiostatic deposition methods^12^. Our PHED method integrates a two-stage chemical process, enabling the use of both electroplating and electrophoretic deposition in a single ‘one pot’ deposition method. In this process, we first dissolve our metal ions and Sn complexing agent (Na_3_C_6_H_5_O_7_) in DI water as electrolyte. Shortly afterwards, we add our Na_2_S_2_O_3_, initiating the formation of a Cu_2−x_S nanoparticle precipitate, in the electrolyte.

The key to preparing a suitable electrolyte lies in the sequence and timing, in which the various precursors are added. The order of addition from first to last is Na_3_C_6_H_5_O_7_ → SnCl_2_ → ZnSO_4_ → CuSO_4_ → Na_2_S_2_O_3_, respectively. There is a specific duration needed before the addition of Na_2_S_2_O_3_ so the complexation reaction between the Na_3_C_6_H_5_O_7_ and Sn^2+^ can be controlled since the reaction duration will affect the elemental composition of the electrodeposited CZTS film.

Figure 1(c) shows the results of the compositional analysis obtained by energy-dispersive X-ray spectroscopy (EDS), and we observe that when the reaction duration is longer, the more complete complexation of the Sn in the electrolyte will hinder the reduction of Sn during the final electrodeposition. Therefore, the relative Sn and Zn percentages decrease while the Cu and S percentages increase. Na_2_S_2_O_3_ is a common sulfur source used in CZTS electrodeposition, and here it is also used as a reducing agent for the Cu. After the oxidation-reduction reaction between copper sulfate and sodium thiosulfate, the Cu^2+^ ions are reduced to 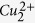 ions and the Cu_2−x_S then precipitates^13^.

First, Cu^2+^ will be reduced by thiosulfate by the reaction


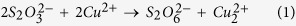


Then, S^2−^ will form by the reactions


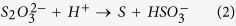



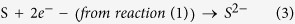


Finally, S^2−^ will react with Cu and precipitate insoluble copper sulfide by


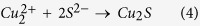


or


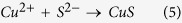


Thus, the color of the electrolyte solution changes from blue to a rusty orange color [Fig. 1(d)].

**Figure 2:**
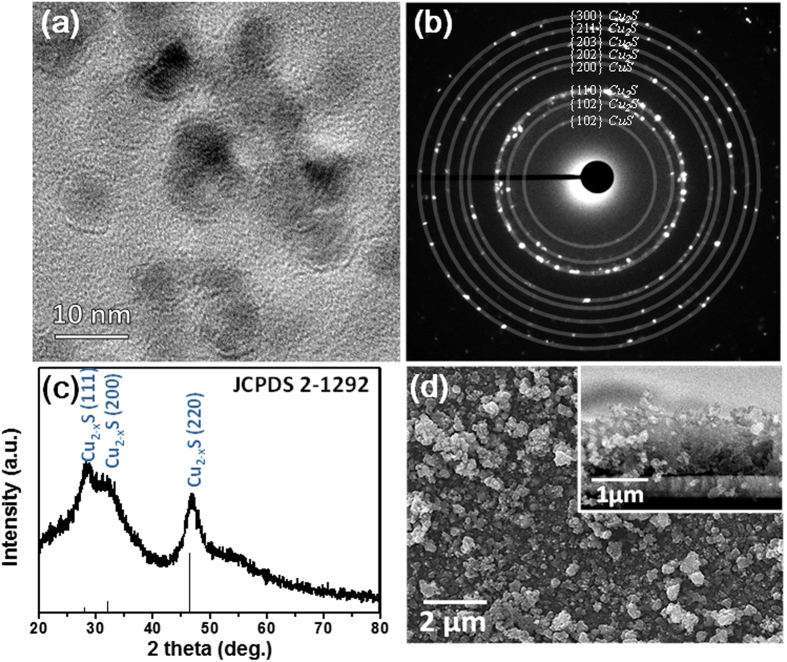
(**a**) TEM image (**b**) SAED pattern and (**c**) XRD spectra of polycrystalline Cu_2−x_S (CuS and Cu_2_S) nanoparticle precipitate prepared in electrolyte solution showing an average diameter of ~10 nm and (**c**) top and cross section (inset) SEM image of PHED-deposited CZTS thin films prior to the sulfurization process.

This oxidation-reduction reaction will precipitate Cu_2−x_S nanoparticles with a diameter of ~10 nm as observed under transmission electron microscopy (TEM) [Fig. 2(a)]. The selective area electron diffraction pattern (SAED) [Fig. 2(b)] can be indexed to the contribution of polycrystalline CuS and Cu_2_S. The X-ray diffraction (XRD) spectra of the precipitate shown in Fig. 2(c) is consistent with the Cu_2−x_S (JCPDS 2-1292 card). The precipitate is primarily composed of Cu (~48.7 at %) and S (~42.5 at %) as observed by EDS (details in Table 1). Once the electrolyte is prepared, the Sn, Zn and Cu_2−x_S were deposited onto the Mo coated SLG substrate using our PHED process. Following 400 cycles of PHED (16:40 min), we obtain a 1 μm thick CZTS thin-film on our Mo coated (400 nm) soda lime glass (SLG) substrate. Top and cross sectional scanning electron microscopy (SEM) images of the CZTS film morphology before sulfurization are shown in Fig. 2(d).

**Figure 3:**
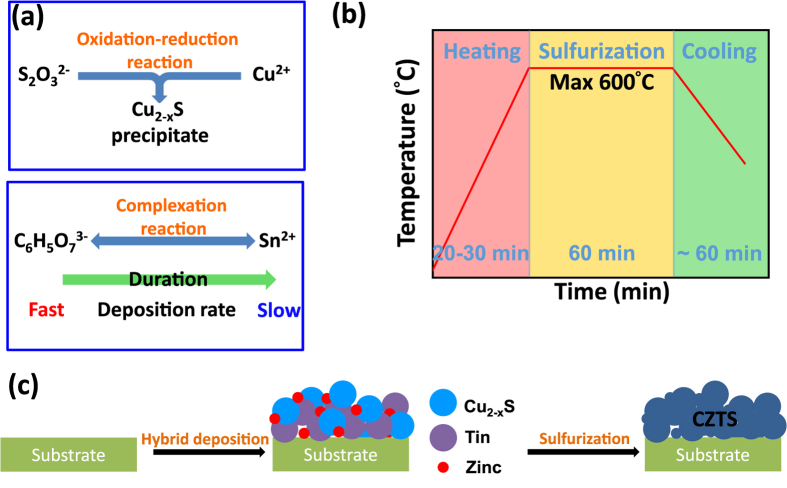
(**a**) Flow diagram illustrations showing (top) evolution of C_2–x_S nanoparticle precipitate formation and (bottom) effect of the duration of complexation reaction between citrate and Sn^2+^ on deposition rate, (**b**) schematic illustration of the sulfurization process timing and (**c**) overall process flow showing PHED film deposition and sulfurization step for the formation of CZTS absorber.

Schematic illustrations of the complexation process for the Sn ions and formation of the Cu_2−x_S nanoparticle precipitate are shown in Fig. 3(a). In order to form a kesterite absorber suitable for solar cell applications, the as-deposited CZTS thin-films must undergo a final sulfurization process whereby the films, along with S and SnS pellets, are placed together in a graphite box, and then placed into a furnace under a H_2_ (20 sccm) and N_2_ (180 sccm) atmosphere at a pressure of 60 torr for 1 hr. The films are heated according to the temperature profile illustrated in Fig. 3(b) across a range of temperatures (450 °C to 600 °C). The high temperature sulfurization process is applied via the following reactions^14^:


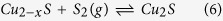



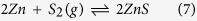



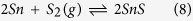



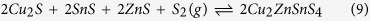


A schematic of the entire film deposition and sulfurization process is shown in Fig. 3(c). During the sulfurization process, the chalcogenides of Cu_2_S, ZnS and SnS will form first, followed by a further reaction with the excess S vapor to form CZTS. Note that the excess S vapor present during the elevated temperature process facilitates the formation of CZTS, while the presence of the SnS pellets helps prevent the loss of Sn^15^,^16^. However, if care is not taken, the equilibrium S vapor pressure will rise as the sulfurization temperature increases^14^, and insufficient S vapor can result in the thermal decomposition of CZTS into secondary phases^17^.

**Figure 4:**
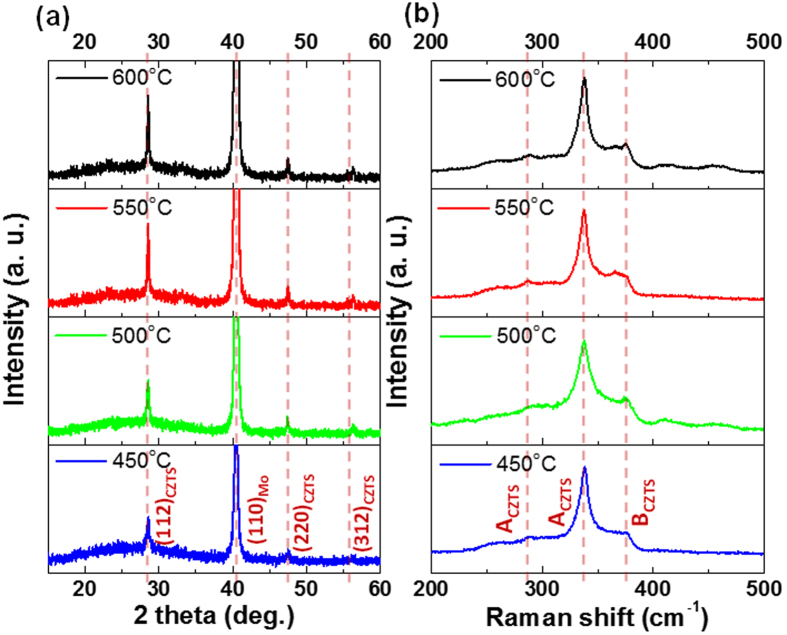
(**a**) XRD and (**b**) Raman spectra of CZTS thin films fabricated using sulfurization process temperatures between 450 °C and 600 °C.

To shed light on the crystallinity of the CZTS, the XRD spectra for each of the films were acquired [Fig. 4(a)] where we can observe three clear peaks corresponding to the (112), (220), and (312) planes of CZTS were indexed. Analysis of the FWHM of the CZTS (112) plane shows that the crystallinity is initially enhanced with increasing sulfurization temperature as summarized in Table 2. Notably, little enhancement is observed when the sulfurization temperature is increased beyond 500 °C. With only a 1.5% decrease of the (112) XRD FWHM observed as we increase the sulfurization temperature from 500 to 600 °C.

The same films with different sulfurization temperatures from 450–600 °C were also examined by Raman spectroscopy as shown in Fig. 4(b) with distinct Raman-active vibration modes at 287, 338 cm^−1^ and B Raman-active vibration modes at 374 cm^−1^ for CZTS found for all sulfurization temperatures^18^. At each sulfurization temperature point, we can clearly observe the expected kesterite related CZTS Raman modes^19^. No significant change was observed in any of the peak intensities or positions with no Cu_2_S, ZnS or SnS related peaks being observed at any temperature, suggesting that sulfurization is being performed within an appropriate process window. Note that various temperatures were investigated with the best performing devices obtained following a 550 °C sulfurization process. The elemental compositions of these ‘best performing’ films pre and post-sulfurization are listed in Table 3 (obtained by EDS analysis), with a post- sulfurization elemental compositions of ~27.18 at %, ~14.44 at %, ~12.13 at %, and ~46.25 at % for Cu, Zn, Sn, and S elements, respectively. In general, it has been observed that moderately Cu-poor and Zn-rich films (such as observed in our pre-sulfurized CZTS) lead to enhanced solar cell performance^20^. In our case, a slight elemental loss was observed during the high temperature sulfurization process, leading to a slight adjustment to our [Cu]/([Zn] + [Sn]) and [Zn]/[Sn] ratios with the [Cu]/([Zn] + [Sn]) ratio being most effected (Table 3). The values represent the smallest changes observed in any of our films prepared at the sulfurization temperature of 550 °C, and correspond to the compositional values reported by others.

**Figure 5:**
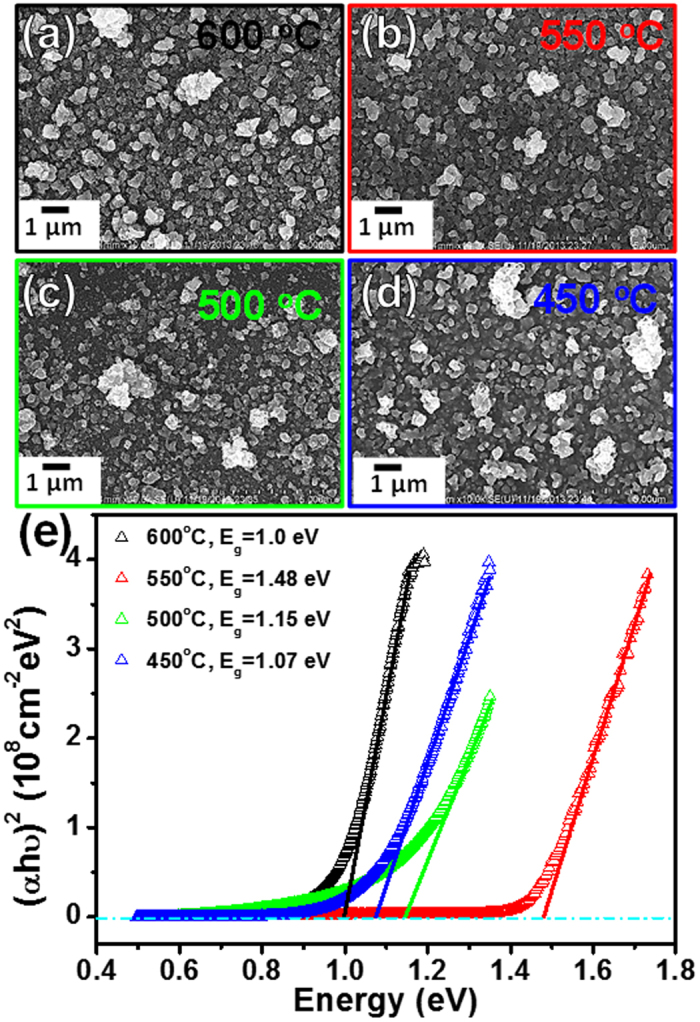
Top view SEM images showing morphologies of CZTS films after the sulfurization processes at (**a**) 600 °C, (**b**) 550 °C, (**c**) 500 °C and (**d**) 450 °C. (**e**) plots of (α*h*υ)^2^ versus different photon energies with extracted optical band-gaps for the CZTS absorber thin-films.

SEM images illustrated in Fig. 5(a–d) reveal similar surface morphologies for all of the CZTS thin-films sulfurized at temperatures from 450 to 600 °C. However, the optical properties of the CZTS thin films measured using UV-Vis spectroscopy vary significantly. The calculated band gap^21^ from the plots of (α*h*υ)^2^ versus incident photon energies in Fig. 5(e) reveal CZTS films sulfurized at 550 °C show the best optical properties in terms of suitability for kesterite solar cell fabrication, with a significantly wider band gap of 1.48 eV^4^. The films sulfurized at relatively lower temperatures of 450 °C and 500 °C reveal smaller band gaps^22^, this may be due to the non-complete formation of kesterite CZTS and the secondary phase formation of Cu_2_SnS_3_^16^,^23^. For the film sulfurized at 600 °C, its reduced band gap is likely due to thermal decomposition of the CZTS as a result of the S vapor being below the equilibrium pressure required to form CZTS^24^.

**Figure 6:**
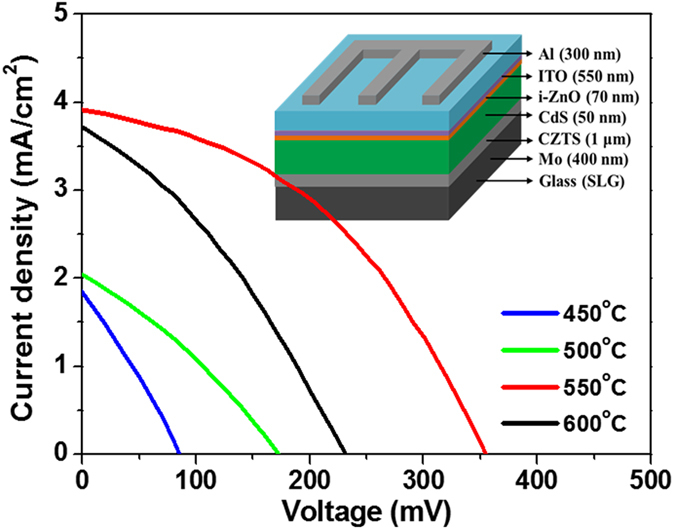
J-V characteristics of PHED CZTS solar cells fabricated using the sulfurization process temperatures between 450 °C and 600 °C measured under air mass 1.5 illumination. Inset shows the device configuration.

To complete the fabrication of the CZTS solar cell devices, we deposited a cadmium sulfide (CdS) buffer layer followed by intrinsic zinc oxide (i-ZnO), an indium doped tin oxide (ITO) layer and a final aluminum electrode (details in the experimental section). The detailed device configuration is shown in inset of Fig. 6 and the corresponding J-V characteristics of CZTS solar cells measured under air mass 1.5 illumination are shown in Fig. 6. The extracted performance data for the complete set of CZTS films is shown in Table 4. Our results show relatively high R_S_ and low R_SH_, resulting in poor current density and open circuit voltage and thereby degradation in overall cell performance. Importantly, the poorer performance of the cells fabricated utilizing the 450 °C, 500 °C and 600 °C sulfurization processes can be attributed to the poor CZTS absorber quality as determined from a combination of the optical band gap and XRD measurements.

Clearly, the cells prepared using a 550 °C sulfurization process exhibit markedly higher performance with a V_OC_, J_SC_, FF and η of 350 mV, 3.90 mA/cm^2^, 0.43 and 0.59%, respectively. The significantly higher R_SH_ observed in these devices is one contributing factor to their enhanced performance, and is likely a consequence of the optimum sulfurization process. The wider band gap of the films prepared using the 550 °C sulfurization process is expected to be the biggest factor in enhancing their outright performance. However, we expect these both of these factors to be inter-related. We believe the remaining cells performances are inhibited by insufficient sulfurization of the CZTS at 450 °C, 500 °C and the onset of thermal decomposition of CZTS at 600 °C as discussed previously. Table 5 lists the progress of quaternary electrodeposition of CZTS. In our work, we demonstrate a facile electrolyte solution preparation method, where in comparison to conventional electroplating methods performed, a reduction in the number of complexing agents is required. As such, we can potentially reduce the complexity and cost of the CZTS absorber fabrication while concurrently increasing the speed at with the films can be deposited. One part of our method that is still open to further enhancement is the optimization of the sulfurization process in order to enhance the cell performance. Specifically, it would be beneficial if we could afford better control of the required S vapor during the elevated sulfurization temperature. We consider this a potential avenue toward achieving further enhanced kesterite CZTS solar cells performance, and will be the subject of future work. Finally, we would like to add that our PHED process is an extremely low-cost process when compared to costly vacuum based thin-film solar cell fabrication methods, and as such offers a viable route toward low-cost sustainable energy.

## Conclusions

In summary, a one-step pulsed hybrid electrophoretic & electroplating deposition method was demonstrated for the deposition of kesterite CZTS thin-films, enabling the formation of high quality films with appropriate elemental compositions suitable for solar cell applications. A facile electrolyte solution preparation method was proposed where in comparison to conventional electroplating methods, a reduction in the number of complexing agents required in the solution at the time of deposition provides a more stable electrolyte environment for film formation, in turn leading to highly uniform films. Likewise, we remove the complexity traditionally associated with nanoparticle preparation required for CZTS electrophoretic deposition. We have investigated the effect of the sulfurization temperature on the films structural and optical qualities, and are able to observe that films exposed to a 550 °C sulfurization process possess the most suitable characteristics for solar cell fabrication. A significant widening of the optical bandgap is observed in films prepared at this temperature, leading to enhanced performance, with our best CZTS solar cell devices having a V_OC_, J_SC_, FF and η of 350 mV, 3.90 mA/cm^2^, 0.43 and 0.59%, respectively. Furthermore, our PHED process is an extremely low-cost process compared to costly vacuum based thin-film solar cell fabrication methods, offering a viable route toward low-cost sustainable energy.

## Methods

The PHED deposition of CZTS thin-films was carried out using a three-electrode cell, consisting of an Mo coated soda lime glass substrate as the working electrode (WE), a graphite bar as the counter electrode (CE) and a silver/silver chloride (Ag/AgCl) electrode as the reference electrode (RE). An electrolyte solution of deionized (DI) water contains 0.0022 M copper sulfate (CuSO_4_), 0.011 M zinc sulfate (ZnSO_4_), 0.005 M tin chloride (SnCl_2_), 0.08 M sodium thiosulfate (Na_2_S_2_O_3_) and 0.05 M tri-sodium citrate (Na_3_C_6_H_5_O_7_) used as a complexing agent for the Sn^2+^ ions, respectively. The pH value of electrolyte was 5.81 with no further pH adjustment applied. To deposit the CZTS films, the pulsed electrodeposition method was applied using a 10% duty cycle (on-time duration of 0.25 sec at −3 V, off-time duration of 2.25 sec at 0 V). The set potential of 0030 V during the off-time is the potential difference between applied voltage and open circuit voltage, which is the steady state without any disturbance of applied potential. Surface morphologies were examined using a Hitachi S-8010 field-emission scanning electron microscope (FE-SEM). Raman spectra for the CIGS films were obtained using a using a HORIBA Jobin-Yvon, LabRAM HR800 system, equipped with a 632.8 nm laser. The quality and crystal structure of the films were confirmed by X-ray diffraction (XRD) using a Shimadzu XRD-6000 with Cu Kα radiation (0.154 nm). Optical reflectance was measured using a Hitachi U-4100 UV–Visible-NIR Spectrometer. The current-voltage characteristics were measured using a Keithley 4200-SCS parameter analyzer under AM 1.5 G solar simulator at a constant temperature of 25 °C.

## Additional Information

**How to cite this article**: Tsai, H.-W. *et al*. Facile Growth of Cu_2_ZnSnS_4_ Thin-Film by One-Step Pulsed Hybrid Electrophoretic and Electroplating Deposition. *Sci. Rep*. **6**, 19102; doi: 10.1038/srep19102 (2016).

## Figures and Tables

**Table 1:** Elemental composition of Cu_2−x_S nanoparticle precipitate in electrolytic solution.

Cu (at%)	Zn (at%)	Sn (at%)	S (at%)
48.74	3.12	5.60	42.54

**Table 2:** XRD FWHM of CZTS (112) plane under varied sulfurization temperatures.

Sulfurization temperature	(112)_FWHM_ (deg.)
450 °C	0.545
500 °C	0.408
550 °C	0.406
600 °C	0.402

**Table 3:** Elemental composition of PHED CZTS thin-films before and after sulfurization at an optimized temperature of 550 °C.

	Cu(at%)	Zn(at%)	Sn(at%)	S (at%)	[Cu]/([Zn]+[Sn])	[Zn]/[Sn]	S/metal
As-deposited	25.33	19.44	14.56	40.67	0.75	1.34	0.69
Sulfurized	27.18	14.44	12.13	46.25	1.02	1.19	0.86

**Table 4:** Photovoltaic performance results for CZTS at different sulfurized temperatures

Sulfurization temperature	V_oc_ (mV)	J_sc_ (mA/cm^2^)	FF	η (%)	R_s_ (Ω cm^2^)	R_sh_ (Ω cm^2^)
450 °C	90	1.83	0.27	0.04	42.25	13.37
500 °C	170	2.03	0.31	0.11	67.98	55.36
550 °C	350	3.90	0.43	0.59	51.10	385.30
600 °C	230	3.70	0.33	0.28	46.52	90.67

**Table 5:** Comparison of quaternary electrodeposition of CZTS thin-films.

Publishedyear	Method	Sulfurizationtemperature (°C)	Efficiency(%)	Ref.
2010	Electroplating	550 °C	N/A	25
2011	Electroplating	550 °C	N/A	26
2011	Electroplating	500 °C	N/A	27
2011	Electroplating	550 °C	N/A	28
2012	Electroplating	550 °C	1.21	29
2013	Electroplating	200-600 °C	N/A	30
2013	Electroplating	500 °C	N/A	31
2013	electrophoretic deposition	Not mentioned	N/A	7
2014	Electroplating	450–580 °C	N/A	32
2014	Electroplating	300–500 °C	N/A	33
2014	Electroplating	550 °C	N/A	34
2014	Electroplating	580 °C	1.66	35
2014	Electroplating	590 °C	5.53	36
2015	Hybrid electrodeposition	450–600 °C	0.59	This work
